# 2,5-Bis(1,1,3,3-tetra­methyl­butyl)thio­phene

**DOI:** 10.1107/S1600536808037434

**Published:** 2008-11-20

**Authors:** Hassan Y. Elnagar, Mahmood Sabahi, Vince J. Gatto, Frank R. Fronczek

**Affiliations:** a Albemarle Process Development Center, Albemarle Corporation, PO Box 341, Baton Rouge, LA 70821, USA; b Department of Chemistry, Louisiana State University, Baton Rouge, LA 70803-1804, USA

## Abstract

There are two independent mol­ecules in the asymmetric unit of the title compound, C_20_H_36_S. Crystals are non-merohedrally twinned by twofold rotation about [001]. The bulky octyl groups of each mol­ecule are on the same side of the thio­phene plane and are approximately parallel. S—C distances are in the range 1.729 (4)–1.745 (3) Å, and the C—S—C angles are 92.98 (18) and 93.08 (17)°. The CH_2_ groups of the octyl groups are involved in weak C—H⋯S intra­molecular inter­actions.

## Related literature

For previous synthetic work, see: Kutz & Corson (1946 ▶); Caeser (1948 ▶). For the catalyst system, see: Elnagar *et al.* (2006 ▶). For a related structure, see: Krebs *et al.* (1992 ▶). For a description of the Cambridge Strucural Database, see: Allen (2002 ▶).
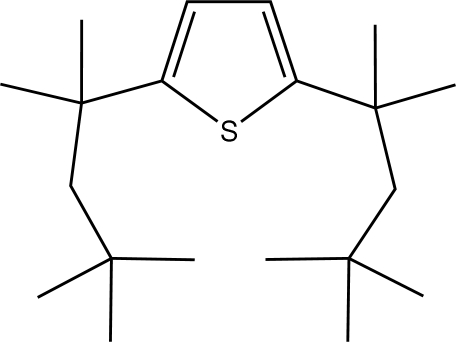


## Experimental

### 

#### Crystal data

C_20_H_36_S
*M* *_r_* = 308.55
Monoclinic, 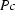

*a* = 21.2367 (6) Å
*b* = 7.9954 (2) Å
*c* = 11.7987 (3) Å
β = 105.059 (2)°
*V* = 1934.57 (9) Å^3^
*Z* = 4
Cu *K*α radiation
μ = 1.40 mm^−1^
*T* = 90.0 (5) K
0.20 × 0.15 × 0.10 mm

#### Data collection

Bruker Kappa APEXII diffractometer
Absorption correction: multi-scan (*SADABS*; Sheldrick, 2004 ▶) *T*_min_ = 0.766, *T*_max_ = 0.872
9297 measured reflections
9297 independent reflections
9129 reflections with *I* > 2σ(*I*)
*R*_int_ = 0.029

#### Refinement

*R*[*F* ^2^ > 2σ(*F* ^2^)] = 0.070
*wR*(*F* ^2^) = 0.182
*S* = 1.08
9297 reflections
403 parameters
2 restraints
H-atom parameters constrained
Δρ_max_ = 1.11 e Å^−3^
Δρ_min_ = −0.42 e Å^−3^
Absolute structure: 2248/609 Friedel pairs corresponding to each component of the non-merohedral twin.

### 

Data collection: *APEX2* (Bruker, 2006 ▶); cell refinement: *SAINT* (Bruker, 2006 ▶); data reduction: *SAINT* and *ROTAX* (Cooper *et al.*, 2002); program(s) used to solve structure: *SHELXS97* (Sheldrick, 2008 ▶); program(s) used to refine structure: *SHELXL97* (Sheldrick, 2008 ▶); molecular graphics: *ORTEP-3 for Windows* (Farrugia, 1997 ▶) and *CrystMol* (Duchamp, 2005 ▶); software used to prepare material for publication: *SHELXTL* (Sheldrick, 2008 ▶).

## Supplementary Material

Crystal structure: contains datablocks global, I. DOI: 10.1107/S1600536808037434/fb2113sup1.cif

Structure factors: contains datablocks I. DOI: 10.1107/S1600536808037434/fb2113Isup2.hkl

Additional supplementary materials:  crystallographic information; 3D view; checkCIF report

## Figures and Tables

**Table 1:** Hydrogen-bond geometry (Å, °)

*D*—H⋯*A*	*D*—H	H⋯*A*	*D*⋯*A*	*D*—H⋯*A*
C8—H8*B*⋯S1	0.99	2.82	3.301 (4)	111
C16—H16*A*⋯S1	0.99	2.78	3.243 (4)	109
C28—H28*A*⋯S2	0.99	2.72	3.209 (4)	111
C36—H36*A*⋯S2	0.99	2.86	3.312 (4)	108

**Fig. 1.**
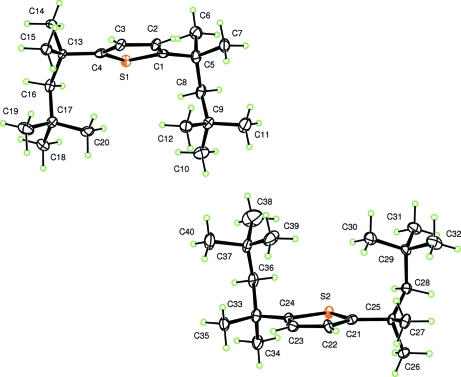
The molecular structure of the title molecules. The displacement ellipsoids shown at the 50% probability level. The H atoms are shown with arbitrary radius.

**Fig. 2.**
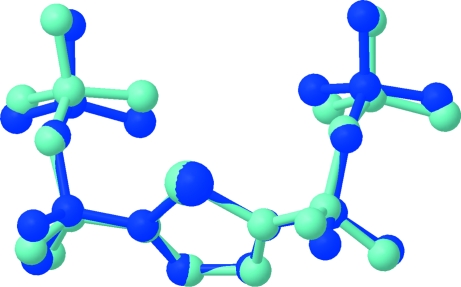
Overlay of two independent molecules of the title structure (Duchamp, 2005). The H atoms are not shown.

## supplementary crystallographic information

### Comment

Kutz & Corson (1946) first reported acid catalyzed alkylation of thiophene with olefins and alcohols. They suggested that the mono-alkylation reaction occurred at the 2-position of the thiophene ring. Higher boiling liquid products were also isolated, which they believed to be di-alkylated thiophenes. Caeser (1948) synthesized 2,5-di-(1,1,3,3-tetramethylbutyl)thiophene by reacting diisobutylene with thiophene and isolated it as a low-melting solid (b.p. 146–147°C, m.p. 36–37°C). He correctly proposed the structure from its physical properties as well as from the work by Kutz & Corson (1946) on the mono-alkyl derivatives.

The two independent molecules of the asymmetric unit are shown in Fig. 1. Their conformations are quite similar, having both octyl chains on the same side of the thiophene plane. A least-squares fit, overlaying the thiophene and three central C atoms of the octyl groups yields an average deviation (11 atoms) of 0.182 Å, as shown in Fig. 2 (Duchamp, 2005). The thiophene rings in both molecules exhibit maximum deviation 0.011 (4) Å from planarity; it is pertinent for C2 in one molecule and C23 in the other. Some geometric features of the thiophene rings are given in Abstract. The molecules deviate slightly from mirror symmetry, as described by the S—C—C—C torsion angles about the bonds attaching the octyl groups to the thiophenes. Torsion angle magnitudes are about 12° larger on one side of the molecule than on the other: S1—C1—C5—C8 = -59.2 (4) and S2—C24—C33—C36 = -57.1 (4)° *vs*. S1—C4—C13—C16 = 47.8 (4) and S2—C21—C25—C28 = 44.7 (4)°.

No otherwise unsubstituted thiophenes having tertiary C atoms adjacent to both S atoms are present in the Cambridge Structural Database (Version 5.29 of November 2007; Allen, 2002) except for several macrocyclic molecules: AMADIB, FOJPOK, FOJPUK, FOJQAX, FOJQEB, LOHVEJ, XEMDOJ, XEMFAX, and YOZHEA. The structure of tetra-t-butylthiophene has been reported (Krebs *et al.*, 1992). It has its thiophene twisted out of planarity as a result of the four bulky substituents.

Weak intramolecular C—H···S interactions involving the CH_2_ groups of the octyl substituents and the thiophene S atoms are listed in Tab. 1. The C—H···S angles are quite small for this type of interaction, near the tetrahedral angle.

### Experimental

Thiophene (42.4 g, 0.50 mol) and diisobutylene (60.8 g, 0.54 mol) were mixed at 20°C, and then the catalyst system containing triethylaluminum (30 ml, 1.0 *M* solution in heptane, 0.03 mol)/hydrogen chloride (120 ml, 1.0 *M* solution in ether, 0.12 mol) was carefully added to control the exothermic reaction, under a nitrogen atmosphere (Elnagar *et al.*, 2006). The resulting reaction mixture was heated for 1 h at 80° C. After workup with 12% NaOH solution, the crude product (67.2 g, 87% yield based on diisobutylene) was obtained as a liquid. It solidified upon standing at room temperature. The solidified material was recrystallized from 20% aqueous 2-propanol to obtain colorless plate-like crystals with a melting point range of 36.9–38.1° C. ^1^H NMR (CDCl_3_) δ 6.57 (s, 2 H), 1.72 (s, 4 H), 1.42 (s, 12 H), 0.86 (s, 18 H); ^13^C NMR (CDCl_3_) δ 154.4, 121.3, 58.3, 38.7, 33.1, 32.8, 31.8.

### Refinement

Though all the H atoms were observable in the difference electron density map, they were situated into the idealized positions. The C—H distances were 0.95 for thiophene C, 0.98 for methyl and 0.99 Å for CH_2_, and thereafter treated as riding. *U*_iso_ for H was assigned as 1.2 × *U*_eq_ of the carrier atoms except for the methyls (1.5). The crystal was a non-merohedral twin with a twinning operation being rotation by 180° about [0 0 1]. The twin law was (-1 0 -0.935, 0 -1 0, 0 0 1), determined by ROTAX (Cooper *et al.*, 2002). (-0.935 ~2a(cosβ)/c.) The number of the reflections in the first and the second domain of the non-merohedral components was 9704 and 2651 respectively. Four domain states were taken into account: two for the non-merohedral components while each moreover had an inversion counterpart. Refinement yielded component proportions 0.80 (2): 0.16 (2) and both inversion-related components 0.02 (2). 2248/609 Friedel pairs were present in the data set for the major/minor component. The largest residual peak was located 1.55 Å from as H20A.

### Crystal data

**Table:**

C_20_H_36_S	*F*_000_ = 688
*M**_r_* = 308.55	*D*_x_ = 1.059 Mg m^−^^3^
Monoclinic, *P**c*	Melting point = 309.9–311.1 K
Hall symbol: P -2yc	Cu *K*α radiation λ = 1.54178 Å
*a* = 21.2367 (6) Å	Cell parameters from 5236 reflections
*b* = 7.9954 (2) Å	θ = 5.5–67.5º
*c* = 11.7987 (3) Å	µ = 1.40 mm^−^^1^
β = 105.059 (2)º	*T* = 90.0 (5) K
*V* = 1934.57 (9) Å^3^	Plate, colourless
*Z* = 4	0.20 × 0.15 × 0.10 mm

### Data collection

**Table:**

Bruker Kappa APEXII diffractometer	9297 independent reflections
Radiation source: fine-focus sealed tube	9129 reflections with *I* > 2σ(*I*)
Monochromator: graphite	*R*_int_ = 0.029
*T* = 90.0(5) K	θ_max_ = 68.0º
φ and ω scans	θ_min_ = 5.5º
Absorption correction: multi-scan(SADABS; Sheldrick, 2004)	*h* = −25→25
*T*_min_ = 0.766, *T*_max_ = 0.872	*k* = −9→9
9297 measured reflections	*l* = −14→14

### Refinement

**Table:**

Refinement on *F*^2^	Secondary atom site location: difference Fourier map
Least-squares matrix: full	Hydrogen site location: difference Fourier map
*R*[*F*^2^ > 2σ(*F*^2^)] = 0.070	H-atom parameters constrained
*wR*(*F*^2^) = 0.182	*w* = 1/[σ^2^(*F*_o_^2^) + (0.0988*P*)^2^ + 3.4793*P*] where *P* = (*F*_o_^2^ + 2*F*_c_^2^)/3
*S* = 1.08	(Δ/σ)_max_ = 0.001
9297 reflections	Δρ_max_ = 1.11 e Å^−^^3^
403 parameters	Δρ_min_ = −0.42 e Å^−^^3^
2 restraints	Extinction correction: SHELXL97 (Sheldrick, 2008), Fc^*^=kFc[1+0.001xFc^2^λ^3^/sin(2θ)]^-1/4^
268 constraints	Extinction coefficient: 0.0051 (6)
Primary atom site location: structure-invariant direct methods	Absolute structure: 2248/609 Friedel pairs corresponding to each component of the non-merohedral twin.

### Special details

**Table:**

Geometry. All e.s.d.'s (except the e.s.d. in the dihedral angle between two l.s. planes) are estimated using the full covariance matrix. The cell e.s.d.'s are taken into account individually in the estimation of e.s.d.'s in distances, angles and torsion angles; correlations between e.s.d.'s in cell parameters are only used when they are defined by crystal symmetry. An approximate (isotropic) treatment of cell e.s.d.'s is used for estimating e.s.d.'s involving l.s. planes.
Refinement. Refinement of *F*^2^ against ALL reflections. The weighted *R*-factor *wR* and goodness of fit *S* are based on *F*^2^, conventional *R*-factors *R* are based on *F*, with *F* set to zero for negative *F*^2^. The threshold expression of *F*^2^ > σ(*F*^2^) is used only for calculating *R*-factors(gt) *etc*. and is not relevant to the choice of reflections for refinement. *R*-factors based on *F*^2^ are statistically about twice as large as those based on *F*, and *R*- factors based on ALL data will be even larger.

### Fractional atomic coordinates and isotropic or equivalent isotropic displacement parameters (Å^2^)

**Table:**

	*x*	*y*	*z*	*U*_iso_*/*U*_eq_	
S1	0.74529 (4)	0.37096 (11)	0.09540 (6)	0.0175 (2)	
S2	0.17907 (4)	1.10233 (10)	0.07647 (7)	0.0163 (2)	
C1	0.68069 (18)	0.2661 (4)	0.1298 (3)	0.0186 (8)	
C2	0.69312 (17)	0.2494 (5)	0.2480 (3)	0.0174 (7)	
H2	0.6645	0.1927	0.2850	0.021*	
C3	0.75267 (17)	0.3243 (5)	0.3119 (3)	0.0175 (7)	
H3	0.7670	0.3249	0.3952	0.021*	
C4	0.78701 (18)	0.3949 (4)	0.2414 (3)	0.0168 (8)	
C5	0.62285 (19)	0.2072 (5)	0.0327 (4)	0.0216 (8)	
C6	0.6476 (2)	0.0883 (5)	−0.0492 (4)	0.0264 (9)	
H6A	0.6753	0.0019	−0.0024	0.040*	
H6B	0.6728	0.1520	−0.0931	0.040*	
H6C	0.6103	0.0355	−0.1043	0.040*	
C7	0.5769 (2)	0.1051 (5)	0.0870 (4)	0.0255 (9)	
H7A	0.6001	0.0074	0.1275	0.038*	
H7B	0.5392	0.0681	0.0250	0.038*	
H7C	0.5620	0.1748	0.1433	0.038*	
C8	0.58894 (19)	0.3537 (5)	−0.0433 (3)	0.0223 (8)	
H8A	0.5605	0.3035	−0.1153	0.027*	
H8B	0.6235	0.4153	−0.0682	0.027*	
C9	0.54724 (18)	0.4873 (5)	−0.0015 (3)	0.0207 (8)	
C10	0.5396 (2)	0.6352 (6)	−0.0882 (4)	0.0327 (10)	
H10A	0.5826	0.6820	−0.0854	0.049*	
H10B	0.5122	0.7216	−0.0664	0.049*	
H10C	0.5191	0.5957	−0.1679	0.049*	
C11	0.4790 (2)	0.4239 (6)	−0.0066 (4)	0.0331 (10)	
H11A	0.4587	0.3812	−0.0855	0.050*	
H11B	0.4527	0.5157	0.0116	0.050*	
H11C	0.4817	0.3338	0.0508	0.050*	
C12	0.5790 (2)	0.5537 (5)	0.1222 (4)	0.0246 (9)	
H12A	0.6225	0.5973	0.1251	0.037*	
H12B	0.5827	0.4626	0.1792	0.037*	
H12C	0.5520	0.6434	0.1414	0.037*	
C13	0.85485 (16)	0.4777 (4)	0.2767 (3)	0.0157 (7)	
C14	0.90328 (19)	0.3590 (5)	0.2417 (4)	0.0194 (8)	
H14A	0.8917	0.3466	0.1562	0.029*	
H14B	0.9017	0.2495	0.2782	0.029*	
H14C	0.9474	0.4051	0.2685	0.029*	
C15	0.87649 (19)	0.4946 (5)	0.4099 (3)	0.0209 (8)	
H15A	0.8439	0.5590	0.4370	0.031*	
H15B	0.9186	0.5525	0.4326	0.031*	
H15C	0.8809	0.3832	0.4457	0.031*	
C16	0.85696 (18)	0.6429 (5)	0.2102 (3)	0.0183 (8)	
H16A	0.8409	0.6164	0.1256	0.022*	
H16B	0.9036	0.6724	0.2238	0.022*	
C17	0.82107 (18)	0.8068 (5)	0.2308 (3)	0.0183 (7)	
C18	0.8267 (2)	0.9278 (5)	0.1335 (4)	0.0235 (8)	
H18A	0.8727	0.9440	0.1360	0.035*	
H18B	0.8072	1.0355	0.1452	0.035*	
H18C	0.8036	0.8814	0.0571	0.035*	
C19	0.8539 (2)	0.8900 (5)	0.3479 (4)	0.0306 (10)	
H19A	0.8420	0.8299	0.4117	0.046*	
H19B	0.8393	1.0065	0.3468	0.046*	
H19C	0.9013	0.8871	0.3602	0.046*	
C20	0.74868 (19)	0.7770 (5)	0.2225 (4)	0.0251 (9)	
H20A	0.7445	0.7036	0.2867	0.038*	
H20B	0.7279	0.7240	0.1472	0.038*	
H20C	0.7274	0.8842	0.2283	0.038*	
C21	0.13402 (18)	1.0878 (4)	0.1789 (3)	0.0151 (7)	
C22	0.16804 (18)	1.1608 (5)	0.2811 (3)	0.0197 (8)	
H22	0.1522	1.1643	0.3492	0.024*	
C23	0.22905 (18)	1.2314 (5)	0.2779 (3)	0.0192 (8)	
H23	0.2569	1.2892	0.3419	0.023*	
C24	0.24308 (17)	1.2070 (4)	0.1732 (3)	0.0158 (7)	
C25	0.06682 (16)	1.0086 (4)	0.1492 (3)	0.0126 (7)	
C26	0.01955 (18)	1.1315 (5)	0.0677 (3)	0.0193 (8)	
H26A	−0.0247	1.0856	0.0486	0.029*	
H26B	0.0332	1.1475	−0.0048	0.029*	
H26C	0.0202	1.2393	0.1076	0.029*	
C27	0.04334 (18)	0.9911 (5)	0.2620 (3)	0.0213 (8)	
H27A	0.0019	0.9301	0.2442	0.032*	
H27B	0.0372	1.1025	0.2921	0.032*	
H27C	0.0760	0.9297	0.3213	0.032*	
C28	0.06434 (18)	0.8429 (5)	0.0803 (3)	0.0168 (7)	
H28A	0.0842	0.8668	0.0148	0.020*	
H28B	0.0177	0.8194	0.0442	0.020*	
C29	0.09494 (18)	0.6761 (4)	0.1364 (3)	0.0171 (7)	
C30	0.1644 (2)	0.6973 (6)	0.2128 (5)	0.0354 (11)	
H30A	0.1635	0.7583	0.2844	0.053*	
H30B	0.1901	0.7602	0.1693	0.053*	
H30C	0.1841	0.5870	0.2337	0.053*	
C31	0.0965 (2)	0.5597 (5)	0.0348 (4)	0.0248 (9)	
H31A	0.0525	0.5492	−0.0173	0.037*	
H31B	0.1123	0.4493	0.0656	0.037*	
H31C	0.1258	0.6059	−0.0092	0.037*	
C32	0.0529 (2)	0.5926 (5)	0.2079 (4)	0.0289 (9)	
H32A	0.0521	0.6631	0.2755	0.043*	
H32B	0.0713	0.4830	0.2355	0.043*	
H32C	0.0084	0.5784	0.1586	0.043*	
C33	0.30216 (19)	1.2630 (5)	0.1341 (3)	0.0195 (8)	
C34	0.2808 (2)	1.3824 (6)	0.0277 (4)	0.0288 (9)	
H34A	0.3194	1.4236	0.0056	0.043*	
H34B	0.2568	1.4771	0.0487	0.043*	
H34C	0.2526	1.3219	−0.0386	0.043*	
C35	0.3491 (2)	1.3595 (5)	0.2330 (4)	0.0247 (9)	
H35A	0.3886	1.3881	0.2089	0.037*	
H35B	0.3608	1.2898	0.3037	0.037*	
H35C	0.3280	1.4622	0.2497	0.037*	
C36	0.33644 (19)	1.1113 (5)	0.0874 (3)	0.0215 (8)	
H36A	0.3025	1.0570	0.0246	0.026*	
H36B	0.3683	1.1606	0.0490	0.026*	
C37	0.37233 (18)	0.9698 (5)	0.1674 (3)	0.0210 (8)	
C38	0.3867 (3)	0.8360 (7)	0.0861 (5)	0.0519 (14)	
H38A	0.4085	0.8869	0.0310	0.078*	
H38B	0.3457	0.7845	0.0422	0.078*	
H38C	0.4150	0.7503	0.1326	0.078*	
C39	0.3342 (2)	0.8904 (6)	0.2456 (5)	0.0410 (12)	
H39A	0.3214	0.9769	0.2942	0.062*	
H39B	0.3615	0.8068	0.2963	0.062*	
H39C	0.2951	0.8363	0.1969	0.062*	
C40	0.4392 (2)	1.0251 (6)	0.2493 (5)	0.0393 (11)	
H40A	0.4319	1.0848	0.3173	0.059*	
H40B	0.4615	1.0990	0.2060	0.059*	
H40C	0.4662	0.9262	0.2761	0.059*	

### Atomic displacement parameters (Å^2^)

**Table:**

	*U*^11^	*U*^22^	*U*^33^	*U*^12^	*U*^13^	*U*^23^
S1	0.0180 (5)	0.0209 (4)	0.0138 (4)	−0.0024 (4)	0.0045 (3)	0.0004 (3)
S2	0.0143 (4)	0.0216 (4)	0.0125 (4)	−0.0029 (4)	0.0026 (3)	−0.0002 (3)
C1	0.0183 (19)	0.0130 (18)	0.0264 (19)	0.0028 (14)	0.0090 (15)	0.0038 (15)
C2	0.0122 (17)	0.024 (2)	0.0171 (17)	0.0004 (14)	0.0055 (13)	0.0010 (14)
C3	0.0132 (17)	0.025 (2)	0.0141 (17)	0.0015 (15)	0.0026 (14)	0.0018 (14)
C4	0.0184 (19)	0.0170 (19)	0.0163 (18)	0.0050 (14)	0.0068 (15)	0.0008 (13)
C5	0.0170 (19)	0.026 (2)	0.024 (2)	−0.0046 (16)	0.0100 (16)	−0.0015 (16)
C6	0.027 (2)	0.027 (2)	0.024 (2)	−0.0017 (17)	0.0055 (17)	−0.0046 (16)
C7	0.018 (2)	0.027 (2)	0.031 (2)	−0.0057 (16)	0.0060 (17)	−0.0036 (16)
C8	0.0159 (19)	0.033 (2)	0.0153 (19)	−0.0036 (16)	0.0000 (15)	−0.0009 (15)
C9	0.0164 (19)	0.026 (2)	0.0194 (19)	0.0024 (16)	0.0041 (15)	0.0017 (15)
C10	0.034 (2)	0.031 (2)	0.034 (2)	0.0077 (18)	0.0114 (19)	0.0005 (18)
C11	0.023 (2)	0.040 (3)	0.034 (2)	0.0006 (18)	0.0042 (18)	−0.0068 (19)
C12	0.023 (2)	0.023 (2)	0.028 (2)	0.0003 (16)	0.0076 (16)	−0.0059 (16)
C13	0.0097 (17)	0.0151 (18)	0.0193 (18)	0.0008 (13)	−0.0018 (13)	0.0009 (14)
C14	0.0189 (19)	0.0124 (18)	0.028 (2)	0.0020 (14)	0.0073 (16)	0.0022 (14)
C15	0.019 (2)	0.020 (2)	0.023 (2)	−0.0028 (15)	0.0052 (15)	0.0050 (15)
C16	0.0153 (18)	0.028 (2)	0.0118 (17)	−0.0026 (15)	0.0041 (14)	0.0023 (14)
C17	0.0168 (18)	0.0195 (19)	0.0182 (18)	0.0014 (15)	0.0041 (14)	0.0000 (14)
C18	0.029 (2)	0.0169 (19)	0.026 (2)	−0.0016 (16)	0.0086 (17)	0.0004 (16)
C19	0.044 (3)	0.021 (2)	0.025 (2)	0.0002 (18)	0.0053 (19)	−0.0029 (16)
C20	0.021 (2)	0.0202 (19)	0.036 (2)	0.0065 (16)	0.0125 (18)	−0.0009 (16)
C21	0.0177 (18)	0.0157 (18)	0.0120 (17)	0.0032 (14)	0.0040 (14)	0.0024 (13)
C22	0.0188 (19)	0.0198 (19)	0.0175 (18)	−0.0089 (15)	−0.0005 (14)	0.0005 (14)
C23	0.0204 (19)	0.0164 (19)	0.0199 (18)	−0.0001 (14)	0.0040 (15)	−0.0009 (14)
C24	0.0138 (17)	0.0168 (18)	0.0153 (17)	−0.0008 (14)	0.0010 (14)	−0.0026 (13)
C25	0.0083 (16)	0.0186 (18)	0.0094 (15)	−0.0010 (13)	−0.0006 (12)	0.0017 (13)
C26	0.0171 (19)	0.0188 (19)	0.0192 (18)	0.0027 (15)	−0.0002 (15)	0.0051 (14)
C27	0.0141 (19)	0.034 (2)	0.0176 (18)	−0.0007 (16)	0.0076 (15)	−0.0002 (16)
C28	0.0149 (18)	0.0193 (19)	0.0151 (17)	−0.0015 (14)	0.0018 (14)	−0.0012 (14)
C29	0.0167 (18)	0.0135 (18)	0.0203 (18)	−0.0030 (14)	0.0036 (14)	0.0049 (14)
C30	0.026 (2)	0.023 (2)	0.048 (3)	−0.0025 (17)	−0.0079 (19)	0.0043 (19)
C31	0.030 (2)	0.019 (2)	0.027 (2)	−0.0021 (17)	0.0121 (18)	0.0012 (16)
C32	0.040 (3)	0.024 (2)	0.030 (2)	−0.0017 (18)	0.0218 (19)	0.0046 (17)
C33	0.0166 (19)	0.026 (2)	0.0138 (18)	−0.0021 (15)	−0.0003 (14)	−0.0020 (15)
C34	0.021 (2)	0.031 (2)	0.034 (2)	−0.0057 (16)	0.0051 (17)	0.0137 (18)
C35	0.019 (2)	0.026 (2)	0.031 (2)	−0.0070 (16)	0.0078 (16)	−0.0029 (16)
C36	0.0164 (19)	0.033 (2)	0.0201 (19)	−0.0083 (16)	0.0144 (15)	−0.0031 (16)
C37	0.0117 (18)	0.028 (2)	0.0233 (19)	−0.0023 (15)	0.0046 (15)	−0.0083 (16)
C38	0.060 (4)	0.054 (3)	0.039 (3)	0.017 (3)	0.008 (3)	−0.020 (3)
C39	0.024 (2)	0.044 (3)	0.061 (3)	0.009 (2)	0.022 (2)	0.029 (2)
C40	0.021 (2)	0.042 (3)	0.048 (3)	−0.009 (2)	−0.005 (2)	0.003 (2)

### Geometric parameters (Å, °)

**Table:**

S1—C4	1.731 (4)	C20—H20B	0.9800
S1—C1	1.743 (4)	C20—H20C	0.9800
S2—C21	1.729 (4)	C21—C22	1.365 (5)
S2—C24	1.745 (3)	C21—C25	1.517 (5)
C1—C2	1.357 (5)	C22—C23	1.423 (5)
C1—C5	1.520 (5)	C22—H22	0.9500
C2—C3	1.425 (5)	C23—C24	1.358 (5)
C2—H2	0.9500	C23—H23	0.9500
C3—C4	1.363 (5)	C24—C33	1.512 (5)
C3—H3	0.9500	C25—C26	1.549 (5)
C4—C13	1.541 (5)	C25—C27	1.544 (5)
C5—C7	1.534 (5)	C25—C28	1.548 (5)
C5—C6	1.542 (5)	C26—H26A	0.9800
C5—C8	1.536 (6)	C26—H26B	0.9800
C6—H6A	0.9800	C26—H26C	0.9800
C6—H6B	0.9800	C27—H27A	0.9800
C6—H6C	0.9800	C27—H27B	0.9800
C7—H7A	0.9800	C27—H27C	0.9800
C7—H7B	0.9800	C28—C29	1.554 (5)
C7—H7C	0.9800	C28—H28A	0.9900
C8—C9	1.548 (5)	C28—H28B	0.9900
C8—H8A	0.9900	C29—C31	1.525 (5)
C8—H8B	0.9900	C29—C30	1.525 (5)
C9—C11	1.522 (6)	C29—C32	1.532 (5)
C9—C12	1.535 (5)	C30—H30A	0.9800
C9—C10	1.544 (6)	C30—H30B	0.9800
C10—H10A	0.9800	C30—H30C	0.9800
C10—H10B	0.9800	C31—H31A	0.9800
C10—H10C	0.9800	C31—H31B	0.9800
C11—H11A	0.9800	C31—H31C	0.9800
C11—H11B	0.9800	C32—H32A	0.9800
C11—H11C	0.9800	C32—H32B	0.9800
C12—H12A	0.9800	C32—H32C	0.9800
C12—H12B	0.9800	C33—C35	1.532 (5)
C12—H12C	0.9800	C33—C34	1.548 (5)
C13—C15	1.525 (5)	C33—C36	1.586 (5)
C13—C14	1.533 (5)	C34—H34A	0.9800
C13—C16	1.542 (5)	C34—H34B	0.9800
C14—H14A	0.9800	C34—H34C	0.9800
C14—H14B	0.9800	C35—H35A	0.9800
C14—H14C	0.9800	C35—H35B	0.9800
C15—H15A	0.9800	C35—H35C	0.9800
C15—H15B	0.9800	C36—C37	1.541 (6)
C15—H15C	0.9800	C36—H36A	0.9900
C16—C17	1.567 (5)	C36—H36B	0.9900
C16—H16A	0.9900	C37—C39	1.516 (6)
C16—H16B	0.9900	C37—C38	1.519 (6)
C17—C19	1.529 (5)	C37—C40	1.559 (6)
C17—C18	1.529 (5)	C38—H38A	0.9800
C17—C20	1.533 (5)	C38—H38B	0.9800
C18—H18A	0.9800	C38—H38C	0.9800
C18—H18B	0.9800	C39—H39A	0.9800
C18—H18C	0.9800	C39—H39B	0.9800
C19—H19A	0.9800	C39—H39C	0.9800
C19—H19B	0.9800	C40—H40A	0.9800
C19—H19C	0.9800	C40—H40B	0.9800
C20—H20A	0.9800	C40—H40C	0.9800
			
C4—S1—C1	92.98 (18)	H20B—C20—H20C	109.5
C21—S2—C24	93.08 (17)	C22—C21—C25	129.3 (3)
C2—C1—C5	130.3 (3)	C22—C21—S2	109.1 (3)
C2—C1—S1	109.4 (3)	C25—C21—S2	121.7 (3)
C5—C1—S1	120.3 (3)	C21—C22—C23	115.0 (3)
C1—C2—C3	114.3 (3)	C21—C22—H22	122.5
C1—C2—H2	122.8	C23—C22—H22	122.5
C3—C2—H2	122.8	C24—C23—C22	112.6 (3)
C4—C3—C2	113.1 (3)	C24—C23—H23	123.7
C4—C3—H3	123.4	C22—C23—H23	123.7
C2—C3—H3	123.4	C23—C24—C33	129.1 (3)
C3—C4—C13	128.6 (3)	C23—C24—S2	110.2 (3)
C3—C4—S1	110.2 (3)	C33—C24—S2	120.7 (3)
C13—C4—S1	121.1 (3)	C21—C25—C26	107.3 (3)
C1—C5—C7	109.1 (3)	C21—C25—C27	109.5 (3)
C1—C5—C6	108.9 (3)	C26—C25—C27	106.9 (3)
C7—C5—C6	106.9 (3)	C21—C25—C28	112.3 (3)
C1—C5—C8	111.6 (3)	C26—C25—C28	106.8 (3)
C7—C5—C8	113.4 (3)	C27—C25—C28	113.7 (3)
C6—C5—C8	106.7 (3)	C25—C26—H26A	109.5
C5—C6—H6A	109.5	C25—C26—H26B	109.5
C5—C6—H6B	109.5	H26A—C26—H26B	109.5
H6A—C6—H6B	109.5	C25—C26—H26C	109.5
C5—C6—H6C	109.5	H26A—C26—H26C	109.5
H6A—C6—H6C	109.5	H26B—C26—H26C	109.5
H6B—C6—H6C	109.5	C25—C27—H27A	109.5
C5—C7—H7A	109.5	C25—C27—H27B	109.5
C5—C7—H7B	109.5	H27A—C27—H27B	109.5
H7A—C7—H7B	109.5	C25—C27—H27C	109.5
C5—C7—H7C	109.5	H27A—C27—H27C	109.5
H7A—C7—H7C	109.5	H27B—C27—H27C	109.5
H7B—C7—H7C	109.5	C25—C28—C29	123.8 (3)
C5—C8—C9	124.1 (3)	C25—C28—H28A	106.4
C5—C8—H8A	106.3	C29—C28—H28A	106.4
C9—C8—H8A	106.3	C25—C28—H28B	106.4
C5—C8—H8B	106.3	C29—C28—H28B	106.4
C9—C8—H8B	106.3	H28A—C28—H28B	106.4
H8A—C8—H8B	106.4	C31—C29—C30	108.5 (3)
C11—C9—C12	109.4 (3)	C31—C29—C32	107.6 (3)
C11—C9—C10	107.1 (4)	C30—C29—C32	109.6 (3)
C12—C9—C10	108.1 (3)	C31—C29—C28	106.3 (3)
C11—C9—C8	112.0 (3)	C30—C29—C28	112.9 (3)
C12—C9—C8	113.3 (3)	C32—C29—C28	111.7 (3)
C10—C9—C8	106.7 (3)	C29—C30—H30A	109.5
C9—C10—H10A	109.5	C29—C30—H30B	109.5
C9—C10—H10B	109.5	H30A—C30—H30B	109.5
H10A—C10—H10B	109.5	C29—C30—H30C	109.5
C9—C10—H10C	109.5	H30A—C30—H30C	109.5
H10A—C10—H10C	109.5	H30B—C30—H30C	109.5
H10B—C10—H10C	109.5	C29—C31—H31A	109.5
C9—C11—H11A	109.5	C29—C31—H31B	109.5
C9—C11—H11B	109.5	H31A—C31—H31B	109.5
H11A—C11—H11B	109.5	C29—C31—H31C	109.5
C9—C11—H11C	109.5	H31A—C31—H31C	109.5
H11A—C11—H11C	109.5	H31B—C31—H31C	109.5
H11B—C11—H11C	109.5	C29—C32—H32A	109.5
C9—C12—H12A	109.5	C29—C32—H32B	109.5
C9—C12—H12B	109.5	H32A—C32—H32B	109.5
H12A—C12—H12B	109.5	C29—C32—H32C	109.5
C9—C12—H12C	109.5	H32A—C32—H32C	109.5
H12A—C12—H12C	109.5	H32B—C32—H32C	109.5
H12B—C12—H12C	109.5	C24—C33—C35	110.1 (3)
C15—C13—C14	106.8 (3)	C24—C33—C34	109.7 (3)
C15—C13—C4	109.0 (3)	C35—C33—C34	107.7 (3)
C14—C13—C4	107.9 (3)	C24—C33—C36	111.8 (3)
C15—C13—C16	114.1 (3)	C35—C33—C36	112.5 (3)
C14—C13—C16	106.4 (3)	C34—C33—C36	104.7 (3)
C4—C13—C16	112.3 (3)	C33—C34—H34A	109.5
C13—C14—H14A	109.5	C33—C34—H34B	109.5
C13—C14—H14B	109.5	H34A—C34—H34B	109.5
H14A—C14—H14B	109.5	C33—C34—H34C	109.5
C13—C14—H14C	109.5	H34A—C34—H34C	109.5
H14A—C14—H14C	109.5	H34B—C34—H34C	109.5
H14B—C14—H14C	109.5	C33—C35—H35A	109.5
C13—C15—H15A	109.5	C33—C35—H35B	109.5
C13—C15—H15B	109.5	H35A—C35—H35B	109.5
H15A—C15—H15B	109.5	C33—C35—H35C	109.5
C13—C15—H15C	109.5	H35A—C35—H35C	109.5
H15A—C15—H15C	109.5	H35B—C35—H35C	109.5
H15B—C15—H15C	109.5	C37—C36—C33	123.2 (3)
C13—C16—C17	123.9 (3)	C37—C36—H36A	106.5
C13—C16—H16A	106.4	C33—C36—H36A	106.5
C17—C16—H16A	106.4	C37—C36—H36B	106.5
C13—C16—H16B	106.4	C33—C36—H36B	106.5
C17—C16—H16B	106.4	H36A—C36—H36B	106.5
H16A—C16—H16B	106.4	C39—C37—C38	108.4 (4)
C19—C17—C18	107.3 (3)	C39—C37—C36	115.0 (3)
C19—C17—C20	109.8 (3)	C38—C37—C36	106.2 (4)
C18—C17—C20	108.8 (3)	C39—C37—C40	106.6 (4)
C19—C17—C16	112.3 (3)	C38—C37—C40	106.9 (4)
C18—C17—C16	106.1 (3)	C36—C37—C40	113.4 (3)
C20—C17—C16	112.3 (3)	C37—C38—H38A	109.5
C17—C18—H18A	109.5	C37—C38—H38B	109.5
C17—C18—H18B	109.5	H38A—C38—H38B	109.5
H18A—C18—H18B	109.5	C37—C38—H38C	109.5
C17—C18—H18C	109.5	H38A—C38—H38C	109.5
H18A—C18—H18C	109.5	H38B—C38—H38C	109.5
H18B—C18—H18C	109.5	C37—C39—H39A	109.5
C17—C19—H19A	109.5	C37—C39—H39B	109.5
C17—C19—H19B	109.5	H39A—C39—H39B	109.5
H19A—C19—H19B	109.5	C37—C39—H39C	109.5
C17—C19—H19C	109.5	H39A—C39—H39C	109.5
H19A—C19—H19C	109.5	H39B—C39—H39C	109.5
H19B—C19—H19C	109.5	C37—C40—H40A	109.5
C17—C20—H20A	109.5	C37—C40—H40B	109.5
C17—C20—H20B	109.5	H40A—C40—H40B	109.5
H20A—C20—H20B	109.5	C37—C40—H40C	109.5
C17—C20—H20C	109.5	H40A—C40—H40C	109.5
H20A—C20—H20C	109.5	H40B—C40—H40C	109.5
			
C4—S1—C1—C2	−1.4 (3)	C24—S2—C21—C22	−0.1 (3)
C4—S1—C1—C5	179.5 (3)	C24—S2—C21—C25	178.2 (3)
C5—C1—C2—C3	−179.0 (4)	C25—C21—C22—C23	−176.8 (3)
S1—C1—C2—C3	2.0 (4)	S2—C21—C22—C23	1.2 (4)
C1—C2—C3—C4	−1.7 (5)	C21—C22—C23—C24	−2.2 (5)
C2—C3—C4—C13	−176.0 (3)	C22—C23—C24—C33	179.4 (4)
C2—C3—C4—S1	0.6 (4)	C22—C23—C24—S2	2.0 (4)
C1—S1—C4—C3	0.4 (3)	C21—S2—C24—C23	−1.2 (3)
C1—S1—C4—C13	177.3 (3)	C21—S2—C24—C33	−178.8 (3)
C2—C1—C5—C7	−4.3 (6)	C22—C21—C25—C26	105.5 (4)
S1—C1—C5—C7	174.7 (3)	S2—C21—C25—C26	−72.4 (3)
C2—C1—C5—C6	−120.7 (4)	C22—C21—C25—C27	−10.2 (5)
S1—C1—C5—C6	58.3 (4)	S2—C21—C25—C27	172.0 (3)
C2—C1—C5—C8	121.8 (4)	C22—C21—C25—C28	−137.4 (4)
S1—C1—C5—C8	−59.2 (4)	S2—C21—C25—C28	44.7 (4)
C1—C5—C8—C9	−71.6 (4)	C21—C25—C28—C29	73.7 (4)
C7—C5—C8—C9	52.1 (5)	C26—C25—C28—C29	−168.9 (3)
C6—C5—C8—C9	169.6 (4)	C27—C25—C28—C29	−51.4 (4)
C5—C8—C9—C11	−79.2 (5)	C25—C28—C29—C31	−166.2 (3)
C5—C8—C9—C12	45.1 (5)	C25—C28—C29—C30	−47.3 (5)
C5—C8—C9—C10	163.9 (4)	C25—C28—C29—C32	76.7 (4)
C3—C4—C13—C15	−8.5 (5)	C23—C24—C33—C35	−0.1 (5)
S1—C4—C13—C15	175.2 (3)	S2—C24—C33—C35	177.0 (3)
C3—C4—C13—C14	107.1 (4)	C23—C24—C33—C34	−118.5 (4)
S1—C4—C13—C14	−69.2 (4)	S2—C24—C33—C34	58.6 (4)
C3—C4—C13—C16	−135.9 (4)	C23—C24—C33—C36	125.7 (4)
S1—C4—C13—C16	47.8 (4)	S2—C24—C33—C36	−57.1 (4)
C15—C13—C16—C17	−55.2 (5)	C24—C33—C36—C37	−68.1 (4)
C14—C13—C16—C17	−172.6 (3)	C35—C33—C36—C37	56.4 (5)
C4—C13—C16—C17	69.6 (4)	C34—C33—C36—C37	173.1 (3)
C13—C16—C17—C19	72.5 (5)	C33—C36—C37—C39	49.9 (5)
C13—C16—C17—C18	−170.7 (3)	C33—C36—C37—C38	169.8 (4)
C13—C16—C17—C20	−51.9 (5)	C33—C36—C37—C40	−73.1 (5)

### Hydrogen-bond geometry (Å, °)

**Table:**

*D*—H···*A*	*D*—H	H···*A*	*D*···*A*	*D*—H···*A*
C8—H8B···S1	0.99	2.82	3.301 (4)	111
C16—H16A···S1	0.99	2.78	3.243 (4)	109
C28—H28A···S2	0.99	2.72	3.209 (4)	111
C36—H36A···S2	0.99	2.86	3.312 (4)	108
